# The Role of Phosphorylated Cx43 on PKC Mediated Ser368 in Lung Injury Induced by Seawater Inhalation

**DOI:** 10.1007/s10753-015-0162-9

**Published:** 2015-04-16

**Authors:** Tonggang Liu, Yanyan Li, Bo Zhang, Lijie Ma, Wei Liu, Zhichao Li, Faguang Jin

**Affiliations:** Department of Respiratory Medicine, Tangdu Hospital, Fourth Military Medical University, Xi’an, Shaanxi 710038 People’s Republic of China; Department of Pathology and Pathophysiology, Fourth Military Medical University, Xi’an, Shaanxi 710032 People’s Republic of China

**Keywords:** seawater, ALI, Connexin43, PKC

## Abstract

Seawater aspiration may result in acute lung injury/acute respiratory distress syndrome (ALI/ARDS), which is characterized by pulmonary inflammation and lung edema that closely related to pulmonary barrier dysfunction and intracellular communication. The aim of the present research was to explore the role of connexion 43 (Cx43) in seawater aspiration-induced ALI/ARDS. The results from *in vivo* experiments showed that seawater inhalation led to increased expression of p-PKC and phosphorylated Cx43 (p-Cx43), which were followed by protein rich fluid leakage and TNF-α and IL-1β secretion. Besides, the results from *in vitro* tests proved that the expression of p-PKC directly influenced phosphorylation state of Cx43 and its function, which could further affect the inflammatory factors secretion and intercellular communication. In conclusion, seawater aspiration causes p-Cx43 expression by PKC pathway, which is involved in the on come and development of pulmonary inflammation and lung edema.

## INTRODUCTION

Seawater aspiration may result in acute lung injury (ALI) and acute respiratory distress syndrome (ARDS), the manifestations of which are up-regulation of inflammatory mediators and appearance of pulmonary edema caused by dysfunctions of barrier function of alveolar epithelial and pulmonary capillary endothelial cells and lung edema [1]. In addition, diffuse confluent alveolar patterns of pulmonary edema could be revealed by chest radiograph 1 h after near drowning in chlorinated water [2]. Seawater may directly damage the alveolar epithelial cells and disrupt the alveolar epithelial barrier (AEB), including gap junctions, which would facilitate fluid transport and inflammatory spreading driven by high osmolality of seawater.

Gap junctions, consisting of connexins (Cxs), are transmembrane channels, which provide direct cytoplasmic continuity and intracellular information exchange between adjacent cells, a process named as gap junctional intercellular communication (GJIC) [3]. And, as a main mode of connection, connexin 43 (Cx43) participates in a variety of acute/chronic lung diseases [4]. It has been established that Cx43 dysfunction appears to influence GJIC and affects the intercellular exchange of small molecules, such as cAMP, cGMP, and Ca^2+^ [5]. These changes may probably result in the disruption of pulmonary permeability and lung edema, which is consistent with our previous results showing seawater stimulation leads to Cx43 expression deregulation, decreased GJIC, and lung injury [6].

Cx43 is synthesized at the rough endoplasmic reticulum (ER), processed at the Golgi apparatus, and ultimately, transported to the plasma membrane as functional protein [5]. Although the available data indicated that the functions of Cx43 could be regulated by internalization, phosphorylation, and degradation, phosphorylation is the most frequently researched pattern [7]. Cx43 can be phosphorylated by several known protein kinases, including protein kinase C, MAP kinase, and the pp60^src^ [8–10]. And the previous evidence showed that decreased phosphorylation of Cx43 at Ser-368 of the C-terminal domain could increase dye permeability in cells exposed to PKC inhibitors [7, 11].

Based on the previous findings, we hypothesize that phosphorylation state of Cx43 mediated by PKC is correlated to pulmonary edema and inflammation. We proved this hypothesis by measuring Cx43, p-PKC, and related inflammatory factors expression in lungs and cells stimulated by seawater.

## MATERIALS AND METHODS

### Animals and Reagents

Male Sprague–Dawley rats, weighing 180–220 g each, were obtained from the Animal Center of the Fourth Military Medical University. The study was approved by the Ethics Review board of the Fourth Military Medical University. All animal experiments were performed in accordance with the National Institute of Health’s guidelines for the care and use of laboratory animals (Publication No.85-23, revised 1985).

The formula seawater was prepared according to the composition of water from the East China Sea provided by Chinese Ocean Bureau: osmolality 1300 mmol/L, pH 8.2, specific gravity 1.05, NaCl 26.518 g/L, MgSO_4_ 3.305 g/L, MgCl_2_ 2.447 g/L, CaCl_2_ 1.141 g/L, KCl 0.725 g/L, NaHCO_3_ 0.202 g/L, and NaBr 0.083 g/L. The artificial seawater was confirmed to be sterile before endotracheal instillation. Anti-p-Ser368 of Cx43, anti-Cx43, anti-phospho-PKC, anti-PKC, and anti-β-actin antibodies for Western blot were purchased from Santa Cruz Biotechnology (Cell Signaling Inc). Staurosporine (STS, inhibitor of PKC) and phorbol myristate acetate (PMA, activator of PKC) were purchased from Beyotime (Nantong, China).

### Modeling and Grouping

The rats were randomly assigned into four groups (*n* = 8 in each): a pre-seawater aspiration group (control), and 1, 4, and 8 h post-seawater aspiration groups (1, 4, and 8 h). The rats were anesthetized by intraperitoneal injection of 3 % pentobarbital sodium (1.5 mL/kg) and anesthesia was maintained by 1 mL/kg every 3 h. The animals were placed in a head-up, 30° supine position on an operating table. A purpose-made catheter was inserted into the left carotid artery to obtain blood samples. A 1 mL syringe was gently inserted into the trachea, approximately 1.5 cm above the carina. Seawater (4 mL/kg body weight) was instilled into both lungs within 4 min. At each time point, arterial blood was sampled for blood gas analysis, and the rats were sacrificed by aortic transection at the end of each time point. The thorax was opened and the right bronchus was ligated before the right lung was isolated.

### Wet-to-Dry Weight Ratio

After the removal of surface blood and fluid, the right upper lobes were weighed (W) immediately, then dried for 72 h at 56 °C in an oven and weighed again (D). The lung wet-to-dry weight ratio (W/D) was calculated as the ratio of the W/D ratio.

### Analysis of Protein in Bronchoalveolar Lavage Fluid

The left lung was lavaged with 1.5 mL of normal saline, the procedure was repeated four times, and the total volume of bronchoalveolar lavage fluid (BALF) recovered was recorded. The protein concentration in BALF was analysed by the Bradford method.

### Measurement of Cytokines

The expression of TNF-α and IL-1β in the lung tissues was determined by using commercially available ELISA kits. Briefly, the lung tissues were homogenized in cool phosphate-buffered saline (lung tissue to PBS 1:5). The assay was carried according to the manufacturer’s instructions.

### Western Blot Analysis

The total proteins (100 μg) were separated on 12 % SDS-polyacrylamide gels and electro-transferred to nitrocellulose membranes. The membranes were incubated overnight with primary antibody (PKC, 1:200; p-PKC, 1:100; p-Cx43, 1:200). After incubation with horseradish peroxidase conjugated secondary antibody (β-actin, 1:5000), the relative levels of the target proteins were determined by chemiluminescence.

### Cell Culture and Treatment

The A549 cells were maintained in 1640-medium supplemented with 10 % fetal calf serum with 100 U/mL of penicillin and 100 μg/mL of streptomycin at 37 °C in humidified atmosphere containing 5 % CO_2_ and 95 % air. The cells were randomly divided into four groups: control group (control), seawater group (SW), PKC inhibitor (SW + STS), and PKC activator (PMA). For the SW group, the cells were exposed to seawater (0.25 mL per 1 mL total volume) for 4 h. The cells in the SW + STS group were pre-treated with STS for 30 min before 4 h seawater stimulation, while the PMA group was treated with PKC activator (PMA) only.

### Scrape-Loading and Dye Transfer Technique

A dye transfer assay was used to evaluate gap junctional communication. A549 cells were rinsed with 2 mL PBS without calcium or magnesium. Two milliliters of 0.075 % LY (molecular weight 443 Da; Sigma, L-0259) dissolved in PBS was added to the cells, and two scrape lines (parallel, equidistant scrapes per well) were made by gently passing a diamond-tipped pen (tip diameter, 0.25 mm) across the cultures. The plates were placed for 5 min at 37 °C in a humidified 5 % CO_2_ incubator. The dye solution was then discarded, and the dishes were rinsed twice with PBS to remove background fluorescence. Monolayers were fixed and permeabilized with 100 % cold acetone at −20 °C for 10 min. Cells were examined with an inverted confocal microscope (FV1000 IX81; Olympus) at emission/excitation wavelengths of 528/425 nm. The images were quantified by counting the number of donor and recipient cells and calculating a cell coupling index with the ratio of recipient to donor cells. The value of dye transfer, defined as the number of secondary recipient cells of LY, was recorded for only one side of the scrape.

### Statistical Analysis

All experiments were performed at least three times. Data are expressed as means ± SD and were analysed by one-way analysis of variance with multiple comparisons. *P* < 0.05 was considered statistically significant.

## RESULTS

### Pulmonary Edema and Microvascular Permeability Induced by Seawater Aspiration

**Fig. 1:**
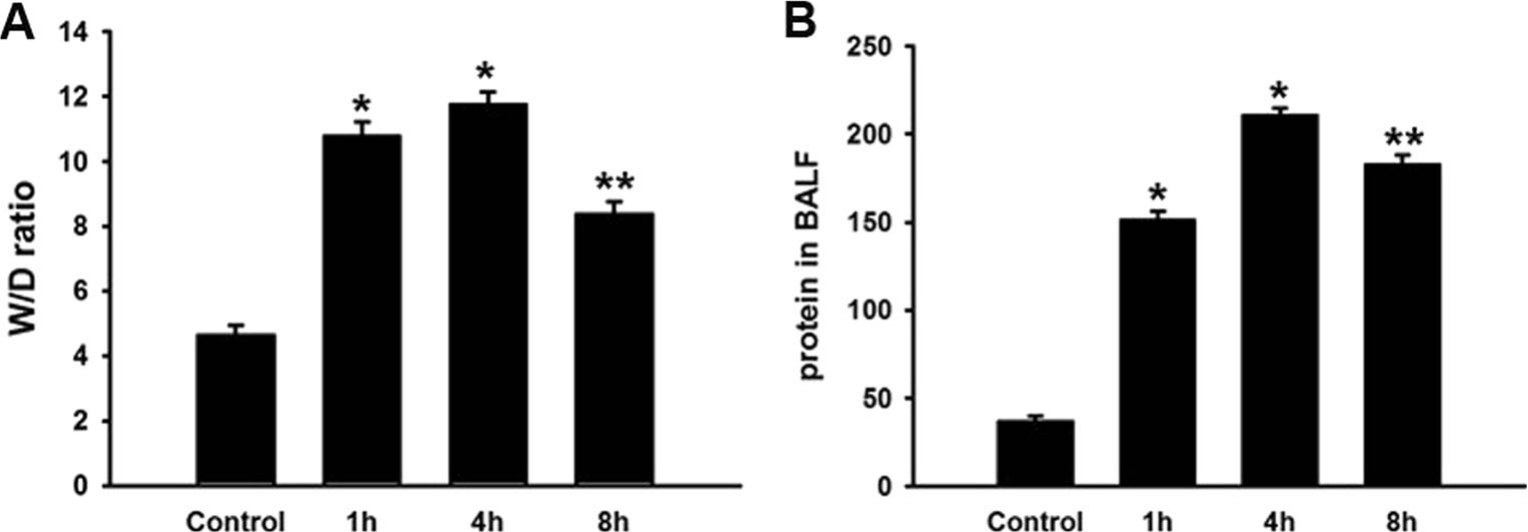
Pulmonary edema and microvascular permeability induced by seawater aspiration. Data are mean ± SD, *n* = 8. ^*^ *P <* 0.01 versus control; ^**^ *P <* 0.05 versus ^*^ *P*.

To assess the lung water accumulation and vascular leakage, the lung W/D ratio was measured. We also detected the protein concentration in BALF (Fig. 1). The lung W/D ratio significantly increased in the rats subjected to seawater aspiration, as compared to the control (*n* = 8, *P* < 0.05). The protein concentration in BALF also markedly increased in the seawater aspiration group compared to the control (*n* = 8, *P* < 0.05).

### Expression of TNF-α and IL-1β in the Lung Tissues and A549 Cells

**Fig. 2:**
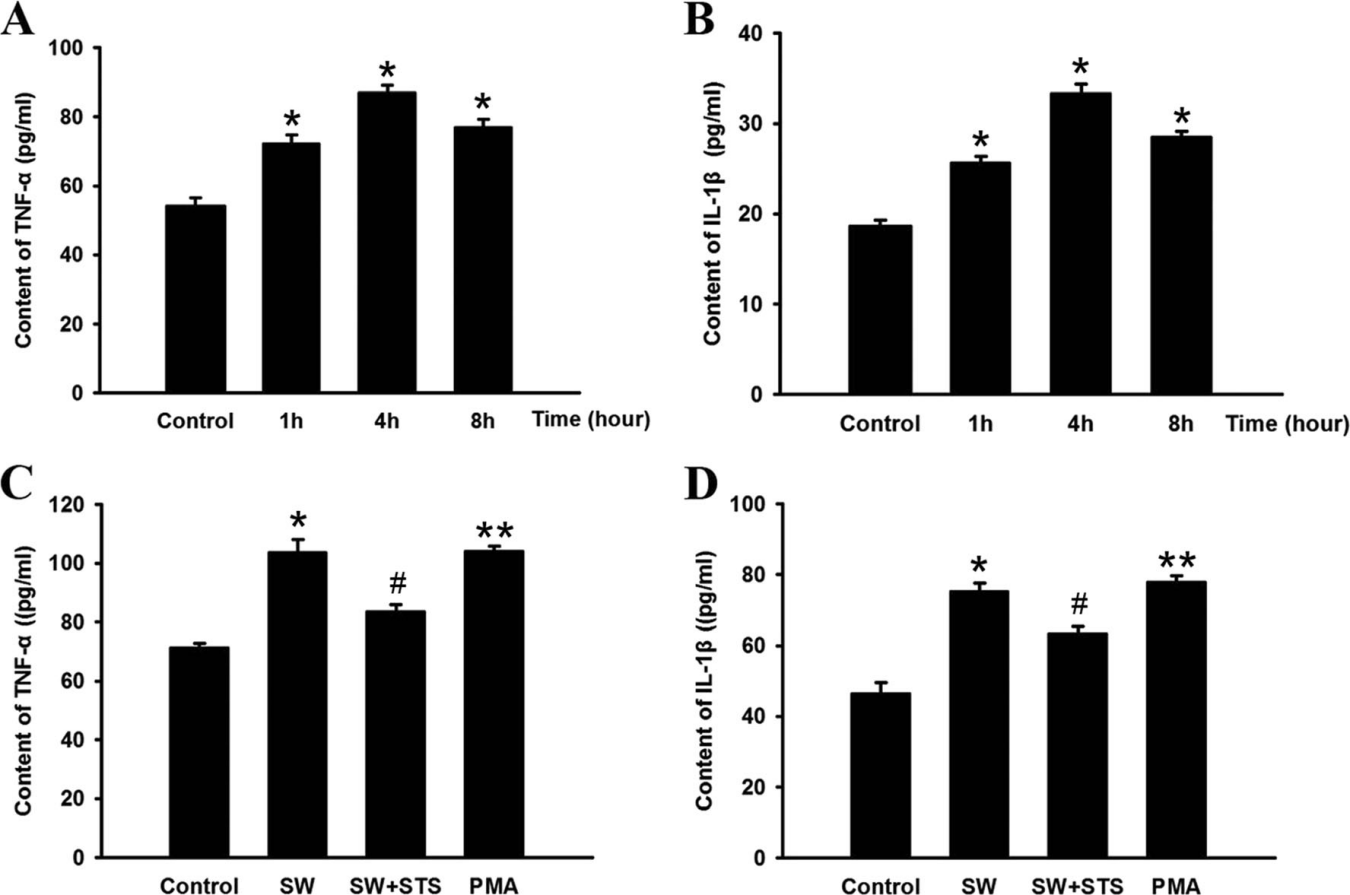
Secretion of TNF-α and IL-1β in lung tissues (**a** and **b**). Data are mean ± SD, *n* = 8. ^*^ *P* < 0.05 versus control; ^*^ *P* < 0.05 versus **P. And TNF-α and IL-1β secretion in seawater stimulated A549 cells (**c** and **d**). ^*#*^ *P < 0.05* versus ^*^ *P*.

We also examine the TNF-α and IL-1β expression in the lungs and A549 cells stimulated by seawater. The results showed that (Fig. 2a, b) seawater inhalation markedly increased TNF-α and IL-1β secretion compared to the control (*n* = 8, ^*^P < 0.05 vs. control) and reached the peak 4 h after stimulation. On the other hand (Fig. 2c, d), TNF-α and IL-1β expression significantly dropped compared to the SW group (^*#*^*P < 0.05* vs. ^*^P) after PKC had been inhibited by STS in A549 cells, and PCK activator alone (MPA) increased TNF-α and IL-1β expression compared to that of control (^**^P < 0.05 vs. control).

### p-PKC Expression in Lungs Stimulated by Seawater

**Fig. 3:**
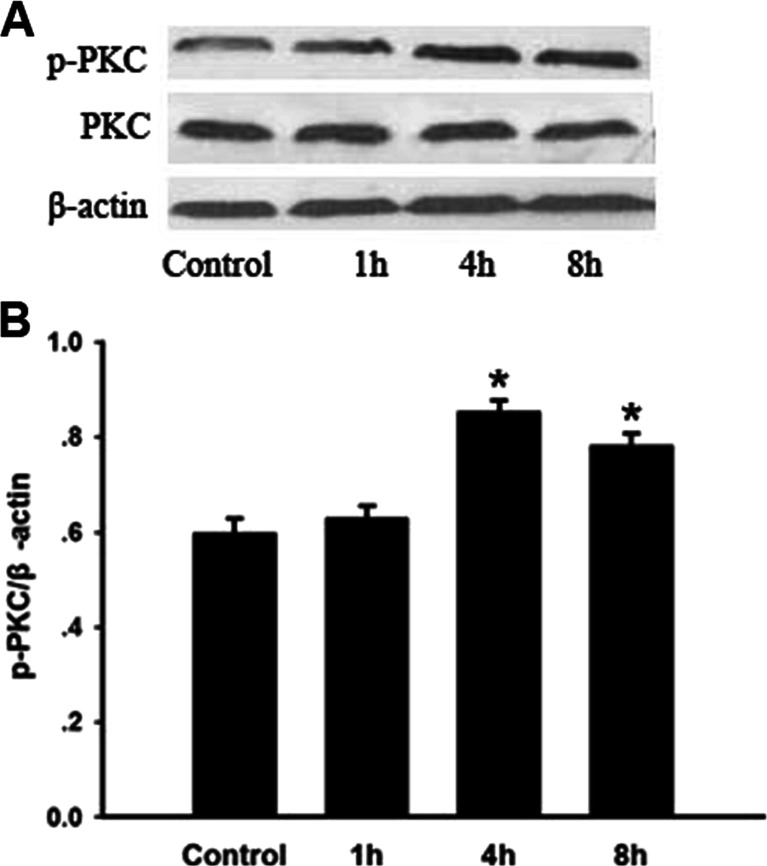
p-PKC expression in lungs stimulated by seawater. After protein quantitation, Western blot was performed and the ratios of p-PKC and PKC versus β-actin were obtained for each group to examine the p-PKC and PKC content. The expression of PKC kept stable after seawater inhalation, while p-PKC expression was significantly increased by seawater, ^*^ *P < 0.05* versus control.

In order to explore the function of PKC, we assessed both the total PKC and p-PKC expressions in lung samples from each group. The total expression of PKC, shown in Fig. 3, was almost stable, while the p-PKC expression in lungs suffered from seawater stimulation increased significantly 4 h after seawater inhalation (^*^P < 0.05).

### Expression of Phosphorylated Cx43 on p-Ser368 in Lung Stimulated by Seawater

**Fig. 4:**
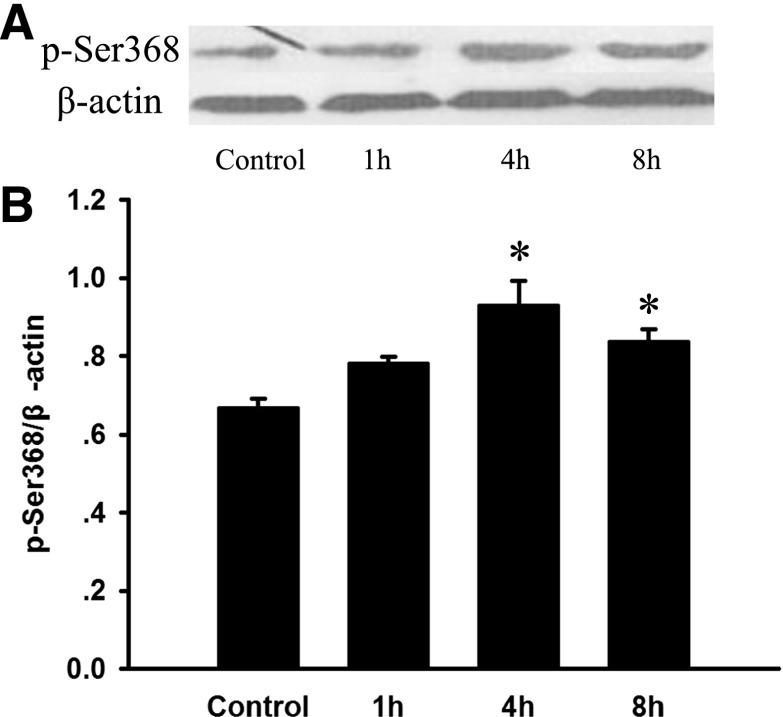
Expression of phosphorylated Cx43 on p-Ser368 in lung stimulated by seawater. After protein quantitation, Western blot was performed and the ratios of phosphorylated Cx43 on p-Ser368 versus β-actin were obtained. The expression of p-Ser368 of Cx43 increased once seawater inhaled into lungs, ^*^ *P <* 0.05 versus control.

To investigate the changes in phosphorylated Cx43 on p-Ser368 after seawater aspiration, we observed the protein levels of p-Ser368 of Cx43 by Western blot (Fig. 4). The results showed that seawater aspiration enhanced the phosphorylation of Cx43 on Ser368. And p-Ser368 of Cx43 levels began to increase after seawater aspiration and peaked at 4 h (^*^P < 0.05).

### Expression of p-PKC in Seawater Stimulated A549 Cells

**Fig. 5:**
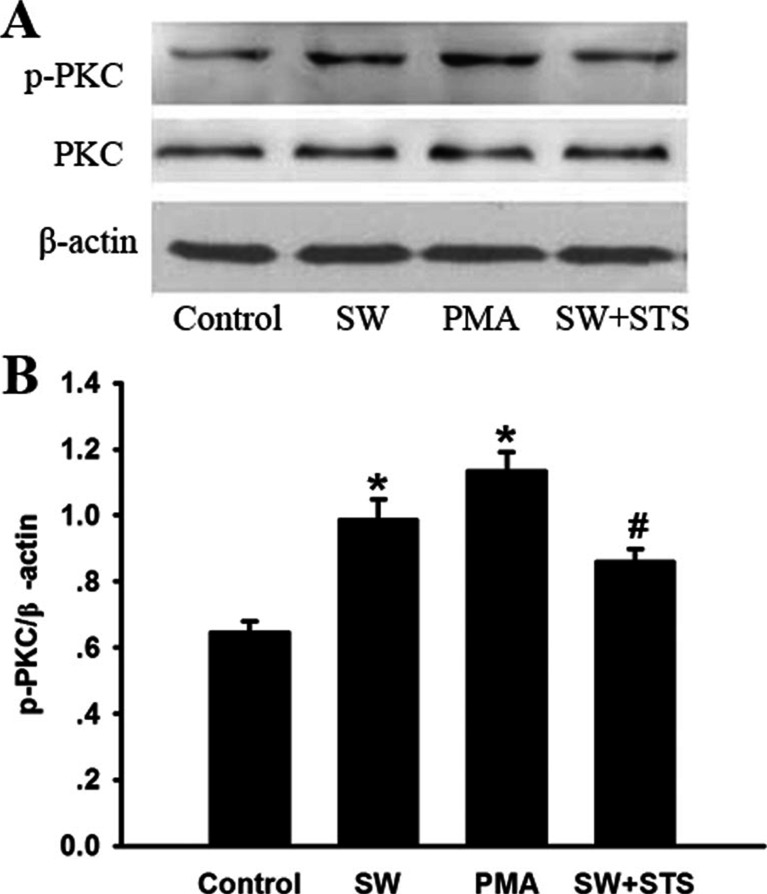
The expression of p-PKC in seawater stimulated A549 cells. After protein quantitation, western blot was performed to evaluate p-PKC expression in A549 cells. Seawater, as well as PMA, markedly increased p-PKC expression ^*^ *P < 0.05* versus control, while inhibitor of PKC (STS) inhibited the expression of p-PKC induced by seawater, ^*#*^ *P < 0.05* versus ^*^ *P*.

The expression of p-PKC in A549 cells exposed to seawater was further evaluated by Western blot (Fig. 5). The results indicated that the phosphorylation of Cx43 increased markedly after seawater exposure at 4 h (^*^P < 0.05), similar to that of activator of PKC (PMA). While the synthetic inhibitor (STS) of PKC reversed the increasing trends of p-PKC induced by seawater (^#^*P* < 0.05 vs. ^*^P)

### Phosphorylation of Cx43 on p-Ser368 in Seawater Stimulated A549 Cells

**Fig. 6:**
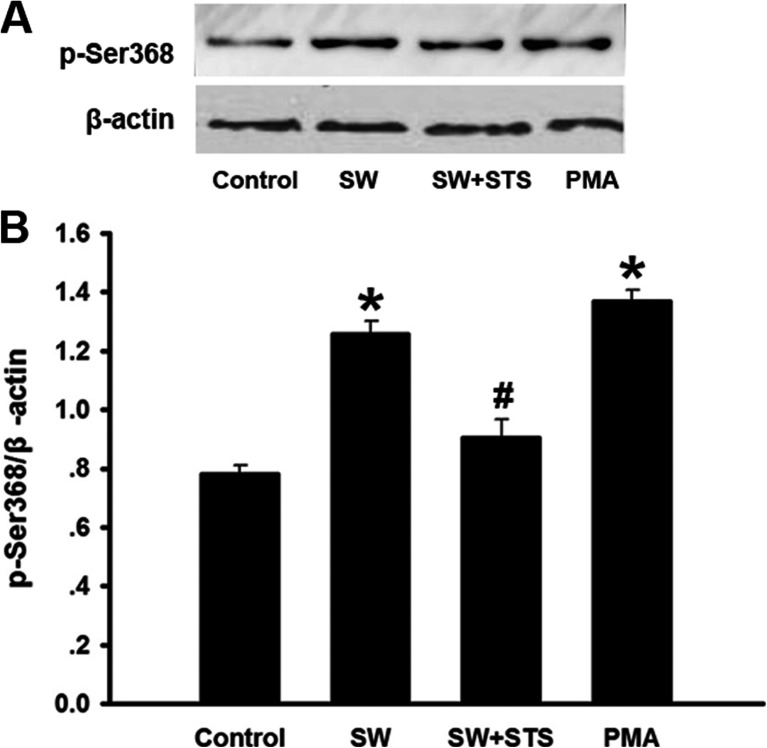
Expression of phosphorylated Cx43 on p-Ser368 in A549cells exposed to seawater. After protein quantitation, western blot was also performed to examine expression of phosphorylated Cx43 on p-Ser368. Seawater and PMA increased p-Ser368 of Cx43 expression^*^ *P <* 0.05 versus control, while inhibitor of PKC (STS) inhibited p-Ser368 of Cx43 expression stimulated by seawater, ^*#*^ *P <* 0.05 versus ^*^ *P*.

In order to further confirm the regulation role of PKC in phosphorylation of Cx43, we evaluated the phosphorylation of Cx43 on p-Ser368 in A549 cells suffered from seawater stimulation (Fig. 6). The results showed that seawater, as well as the PKC activator (PMA), could increase the phosphorylation of Cx43 on Ser368 (^*^P < 0.05), whereas the synthetic inhibitor (STS) of PKC significantly reduced the phosphorylation of Cx43 on p-Ser368 induced by seawater (^#^*P* < 0.05 vs. ^*^P).

### Effects of Seawater on Dye Transfer in A549 Cells

**Fig. 7:**
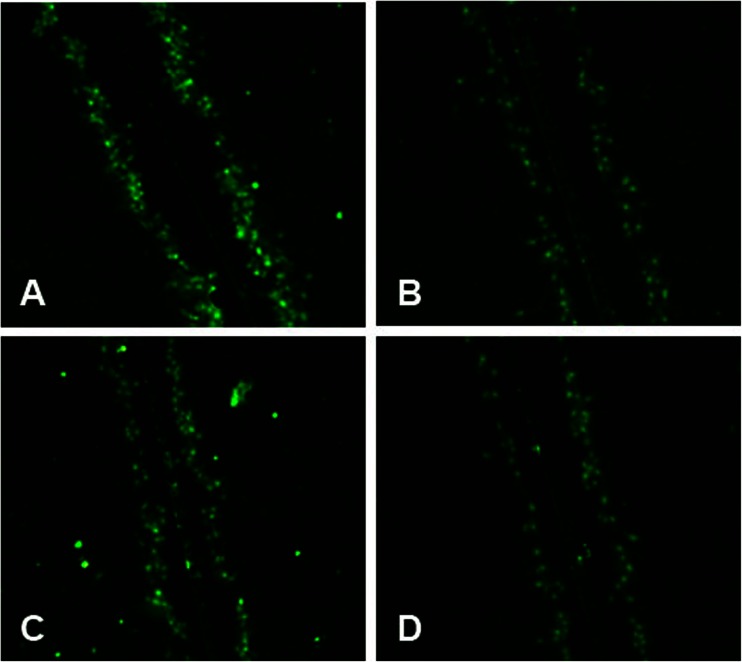
Dye transfer in A549 cells. The panels showed regions of A549 cells on slides scrape-loaded with Lucifer yellow (LY): **a** Control; **b** seawater; **c** seawater + STS; **d** PMA. *Green* indicated LY, including donor cells initially loaded with LY and recipient cells linked together by gap junctional intercellular communication.

**Fig. 8:**
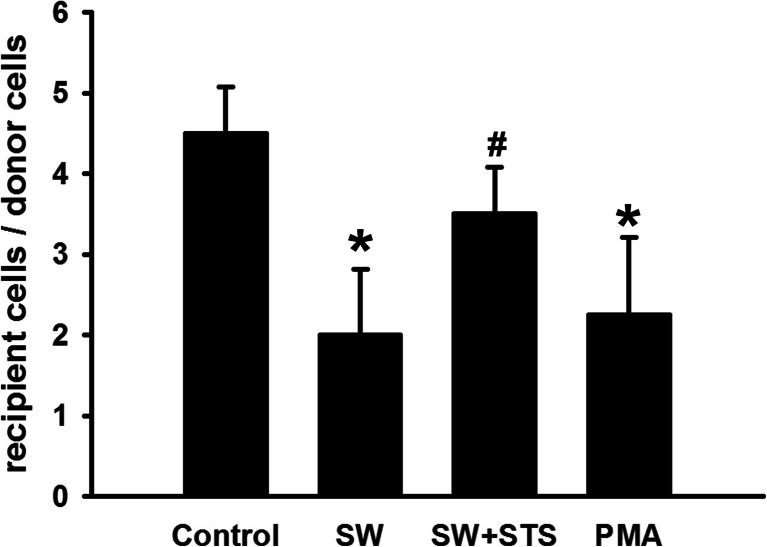
Quantification of dye transfer in A549 cells. ^*^ *P <* 0.05. ^*#*^ *P <* 0.05 versus ^*^ *P*.

A549 cells of similar density were loaded with dye in all groups (Figs. 7 and 8). Compared with the control, seawater stimulation significantly reduced the labeled neighboring cells and reduced the recipient cells to donor cell ratios (^*^P < 0.05), similar to that of PKC activator (PMA), while the pre-treatment of synthetic inhibitor (STS) of PKC markedly enhanced the intracellular exchange of dye and increased the recipient-to-donor ratios (^#^*P* < 0.01 vs. ^*^P).

## DISCUSSION

In the present research, we demonstrated that seawater aspiration led to lung edema and pulmonary inflammation possibly by increasing phosphorylation of Cx43 on Ser368 (p-Ser368). What was more, deregulated p-Ser368 palliated the destruction of the barrier function and relieved the inflammation to some extent both *in vitro* and *in vivo*. Our findings suggested that the phosphorylation of Cx43 on Ser368 was closely related to p-PKC, and this pathway may play a pivotal role in pulmonary edema and inflammation induced by seawater.

As the third most common cause of accidental death [12, 13], drowning has been associated with an increased incidence of ALI, which is similar to sepsis and direct pulmonary injury from lung contusion. The alveolar-capillary membrane would be damaged in ALI, leading to the disruption of epithelial cell barrier function and accumulation of fluid in the alveolar space. This was in accordance with that more than 85 % ARDS patients would suffer from lung edema and lung-fluid clearance defection, which contributes to high levels of morbidity and mortality [14, 15].

Compromised barrier function is a particular concern in the occurrence and development of ALI, and previous researches showed that patients with impaired lung fluid balance were three times more likely to die of ARDS than patients with a normal ability to clear lung fluid [16, 17]. In the present research, the rat models of seawater-induced ALI were established by intratracheal instillation of seawater (4 mL/kg) [18], and the circulating blood volume would be driven into alveolar spaces by hypertonicity. The results of the present research showed that the W/D ratio and the protein concentration in BALF increased after seawater aspiration, indicating that hyperpermeability of alveolar-capillary membrane barrier would facilitate the lung injury induced by seawater stimulation.

Epitheliums are the chief components for barrier function, and alveolar epithelial injury may suppress the coordination and communication between adjacent cells which would lead to a series of complex pathological process [19, 20]. It is well established that the gap junctional communication is directly attributable to cell-cell contact sites. Connexins (Cxs) are constituents of gap junction channels that provide a pathway for the diffusion of ions and small molecules such as cAMP, cGMP, and Ca^2+^ [21]. Cx43 is ubiquitous in both alveolar epithelial and endothelial cells. Numerous evidence indicates that dysfunction of Cx43 is closely related to many kinds of disease [22, 23]. Our previous research demonstrated that the total expression of Cx43 would be suppressed by seawater stimulation [6], and we further explored the effects of phosphorylation site of Cx43 and the regulation role of PKC in the present research.

Phosphorylation of Cx43 has been shown to disrupt intercellular dye transfer, and Ser368 is responsible for the regulation of Cx43 gap junctional hemichannels (GJHs) solute permeability by p-PKC-mediated phosphorylation [24]. The epithelium is the chief component of barrier function and alveolar epithelial injury is sufficient for pulmonary edema formation even in the presence of normal endothelial permeability [19, 20]. We found, in the present research, that p-Ser368 of Cx43 expression increased together with the up-regulated PKC expression in seawater stimulated lungs, being aggrieved by protein rich fluid leaking, and pulmonary inflammation. On the other hand, the *in vitro* experiments, performed on A549 cells, showed that PKC was activated and p-Ser368 of Cx43 expression increased in A549 cells 4 h after seawater stimulation, besides, seawater could not increase phosphorylated Cx43 expression if PKC were inhibited by STS. In addition, since STS has a wide range of biological functions, we also investigated the function of p-PKC by using its inhibitor STS. The results showed that STS could prevent p-PKC from up-regulating phosphorylated Cx43 expression and lead to alleviated lung edema and inflammatory factors secretion.

Seawater inhalation induced acute lung injury (SWI-ALI) is a complicate issue, and the inflammation response has been demonstrated to be crucial during the pathological process [18, 25]. Besides, GJIC between adjacent cells has been reported as a factor contributing to increased pulmonary permeability in lung inflammation [26]. In order to investigate whether Cx43-related GJIC was mediated by the PKC signalling pathway, A549 cells were pre-treated with PKC inhibitor (STS) or activator (PMA), and the results showed that the effects of STS and PMA were totally opposite, and STS could ameliorate phosphorylation of Cx43, reduce the secretion of TNF-α and IL-1β, and inhibit the intracellular communication by inhibiting PKC. More importantly, the expression of phosphorylated Cx43 and function of GJIC would significantly influence secretion of TNF-α and IL-1β in A549 cells. Those findings were confirmed by other researches indicating that dysfunction of GJIC may give rise to many kinds of lung diseases [22, 23].

Phosphorylation of Cx43 mediated by PKC on Ser368 plays a critical role in seawater inhalation induced acute injury by increasing pulmonary permeability, inhibiting intracellular communication, and disturbing the pulmonary barrier. Whereas, seawater induced acute lung injury was a complex process, and this reported mechanism cannot explain it completely. There is a long way to further reveal how this injury happened and what we can do to cure this injury.
